# Hemoglobin-Albumin-Lymphocyte-Platelet (HALP) Score as a Predictive Value of Incidental Prostate Cancer for Patients Going for Transurethral Resection of the Prostate (TURP): A Single-Center Study

**DOI:** 10.7759/cureus.57736

**Published:** 2024-04-06

**Authors:** Ahmed Bendari, Alaa Bendari, Xuelin Zhong, Saroja Devi Geetha, Reham Al-Refai, Sunder Sham, Hanaa Mohammed, FNU Anjali, Pamela Unger

**Affiliations:** 1 Department of Pathology, Lenox Hill Hospital, New York, USA; 2 Department of Medical Biochemistry, Faculty of Medicine, Zagazig University, Zagazig, EGY; 3 Department of Pathology, Zucker School of Medicine, North Shore University Hospital/Long Island Jewish Medical Center, Northwell Health, Greenvale, USA; 4 Department of Human Anatomy and Embryology, Faculty of Medicine, Sohag University, Sohag, EGY; 5 Department of Internal Medicine, Sakhi Baba General Hospital, Sukkur, PAK

**Keywords:** transurethral resection of the prostate, prostate-specific antigen (psa), benign prostatic hyperplasia, halp score, incidental prostate cancer

## Abstract

Aims

Prostate cancer (PC) is a significant health concern worldwide, and early detection is crucial for effective treatment. This study aimed to investigate the role of the hemoglobin-albumin-lymphocyte-platelet (HALP) score in detecting prostate cancer in patients undergoing transurethral resection of the prostate (TURP). Additionally, a comprehensive analysis was performed to explore clinical parameters associated with incidentally diagnosed prostate cancer post TURP.

Methods

A total of 131 patients with symptomatic bladder outlet obstruction who underwent TURP were included in the study. The patients were divided into two groups: those with benign prostatic hyperplasia (BPH) and those with incidental prostate cancer (IPC). The IPC group consisted of patients with both low-grade and high-grade IPC determined by the Gleason score. Demographic data, including age, race, medical history, body mass index, smoking and alcohol status, and family history of prostate cancer, were evaluated. The postoperative measurement of specimen weight and prostate-specific antigen (PSA) levels were also analyzed.

Result

Results revealed that approximately 50% of the patients had BPH, while the remaining 50% had IPC. Patients with IPC, particularly high-grade IPC, had significantly higher PSA levels and lower resected specimen weight compared to those with BPH. The HALP score, which incorporates hemoglobin (Hb), albumin, lymphocyte, and platelet levels, showed promise as a discriminatory tool for distinguishing between BPH and IPC, as well as between high-grade IPC and BPH/low-grade IPC. Logistic regression analysis identified increased PSA levels (p=0.02), decreased HALP score (p≤0.001), and smaller specimen weight (p=0.007) as independent predictive factors for IPC after TURP. Notably, the HALP score was the only significant independent predictive factor associated with high-grade IPC (p=0.004).

Conclusion

These findings contribute to the understanding of risk factors and diagnostic tools for incidentally detected prostate cancer in patients with bladder outlet obstruction undergoing TURP. The HALP score, along with PSA levels and specimen weight, can aid in the early detection and management of prostate cancer. Further research is warranted to validate these findings and explore the clinical utility of the HALP score in predicting prostate cancer outcomes.

## Introduction

Prostate cancer (PC) is the second most frequent cancer diagnosis made in males and the fifth leading cause of death worldwide [1]. Prostate cancer may be asymptomatic at an early stage and often has an indolent course.

Prostate cancer is graded based on the Gleason score system, which determines the aggressiveness of prostate cancer. The score ranges from 2 to 10 and is calculated by adding the grades of the two largest areas of the cancer tissue [2]. The lower the score, the more the cancer cells resemble healthy prostate cells. The higher the score, the more abnormal and likely to spread the cancer cells are. A higher Gleason score is associated with a higher grade of cancer and the likelihood of metastasis [3].

Inflammation and nutrition have been reported to be associated with cancer progression, drug response, and cancer survival. Many inflammatory or nutritional prognostic indices, including the neutrophil-to-lymphocyte ratio (NLR), lymphocyte-to-monocyte ratio (LMR), prognostic nutrition index (PNI), and albumin-to-globulin ratio (AGR), have been developed to predict survival outcomes in solid tumors [4]. The hemoglobin-albumin-lymphocyte-platelet (HALP) score, a novel biomarker that was defined as hemoglobin (Hb)×albumin×lymphocytes/platelets, was first introduced in 2015 by Chen et al. for predicting survival outcomes in patients with gastric cancer. A lower HALP score, which is formulated by hemoglobin×albumin×lymphocytes/platelets, is correlated with worse survival outcomes [4].

In our study, we aim to evaluate the role of the HALP score in detecting PC in patients undergoing transurethral resection of the prostate (TURP). Additionally, we also aim to make a broad comprehensive analysis of patients who are incidentally diagnosed with PC after the TURP procedure, based on clinical parameters such as age, race, past medical history (diabetes and hypertension), body mass index, smoking and alcohol status, family history of prostate cancer, and postoperative measurement of specimen weight.

This article was previously posted to medRxiv preprint server on January 24, 2024.

## Materials and methods

Study design and data collection

**Figure 1:**
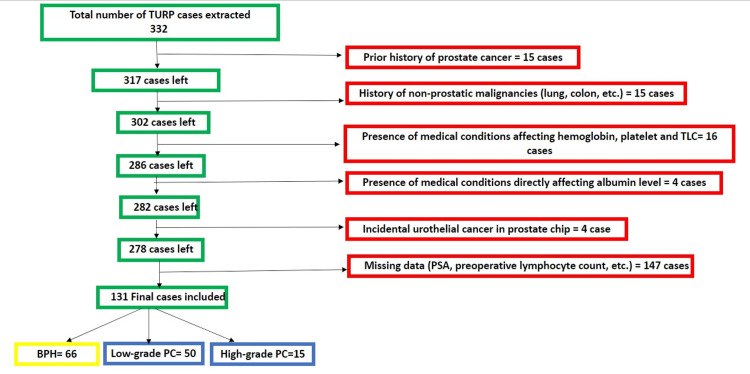
Summary of the patient selection process TURP, transurethral resection of the prostate; PC, prostate cancer; PSA, prostate-specific antigen; TLC, total leukocytic count; BPH, benign prostatic hyperplasia

This study retrospectively utilized our electronic medical record system (Cerner, Kansas City, MO) at Lenox Hill Hospital. The Feinstein Institutes for Medical Research, Northwell Health System, New York City, USA, issued approval 23-071. The study included all patients who underwent TURP between January 2019 and January 2023, meeting specific criteria such as having no significant medical conditions other than benign prostate hyperplasia and being incidentally diagnosed with prostate cancer after histologic examination. Among the 332 patient charts reviewed, a total of 131 cases met the criteria and were included in the analysis. Patients were excluded if they had a prior diagnosis of prostate cancer or any other cancer, if the data (prostate-specific antigen {PSA}, total leucocytic count {TLC}, hemoglobin (Hb), albumin, etc.) were missing, if the patient had conditions that directly affect serum albumin (e.g., liver cirrhosis) or total leucocytic count (TLC) (e.g., leukemia, lymphoma, or active infection before the time), or if the microscopic examination of the TURP prostate tissue was positive for tumor other than prostate cancer (e.g., urothelial cancer). The patient selection process is summarized in Figure 1.

Study variables and HALP, NLR, and PLR score calculations

Chart review was conducted through the hospital's electronic medical record systems (Sunrise {Altera Digital Health, Niagara Falls, NY} and Allscripts {Chicago, IL}) and the pathology database system (Cerner, our and patient data, including age, race, body mass index {BMI}, comorbidities, family history of prostate cancer, smoking and alcohol status, preoperative laboratory factors {PSA, absolute neutrophil count (K/μL), absolute lymphocyte level (K/μL), platelet count (K/μL), hemoglobin level (g/dL), and serum albumin level (g/dL)}, and postoperative data such as specimen weight (g); either tissue was submitted entirely or representatively and the final pathology diagnosis. All preoperative laboratory investigations were done for all patients within the time frame (two days to two weeks before the TURP surgery). HALP, NLR, and PLR scores were calculated as follows [4]: HALP score calculation, [hemoglobin level (g/L)×serum albumin (g/L)×absolute lymphocyte count (K/μL)]/platelet count (K/μL); NLR score calculation, absolute neutrophile count (K/μL)/absolute lymphocyte count (K/μL); and PLR score calculation, platelets count (K/μL)/absolute lymphocyte count (K/μL).

Patient characteristics

We searched our pathology system (Cerner) for keywords related to benign prostatic hyperplasia (BPH), transurethral resection of the prostate, and prostate chips. A total of 332 charts were extracted and manually checked for inclusion criteria, resulting in a final sample size of 131 cases. The exclusion criteria included a prior history of prostate cancer or other malignancies, medical conditions impacting hemoglobin and platelet count, and conditions affecting albumin level, as well as cases with preoperative active infection or fever. Two pathologists have reviewed all cases, and PIN-4 cocktail and P63 immunohistochemistry (IHC) were performed on all equivocal cases.

Specimen: Grossing, tissue submission, and microscopic examination

We followed the College of American Pathologists (CAP) protocol in the submission of prostate tissue, by embedding all first 12 g and then one additional block per each 5 g, and the protocol for TURP specimen grossing and submission: If the specimen weighs less than 12 g, it is submitted entirely for histologic examination; if the specimen weighs more than 12 g, the first 12 g of the tissue is submitted entirely, followed by one cassette of tissue with every additional 5 g of submitted tissue for histologic examination) [2]. PIN-4 cocktail and P63 immunohistochemical stains were performed on all equivocal cases, and all cases were reviewed by two pathologists.

Grading and scoring

We categorized the cases into three groups: benign prostatic hyperplasia, low-grade prostate cancer, and high-grade prostate cancer. The low-grade group is defined as prostate cancer with a Gleason score of 6 (Gleason pattern=3+3) and 7 (Gleason patterns=3+4 or 4+3). The high-grade group is defined as prostate cancer with a Gleason score of 8 (Gleason patterns=4+4 or 3+5 or 5+3), 9 (Gleason patterns=5+4 or 4+5), and 10 (Gleason pattern=5+5) [2].

Statistical analysis

Data analysis was performed using Jamovi (version 2.3, The Jamovi Project, Sydney, Australia). Descriptive statistics such as median (interquartile range {IQR}) and frequency and percentage were reported for continuous and categorical variables, respectively. Nonparametric tests, such as the Kruskal-Wallis and chi-square tests, were used for analyzing continuous and categorical variables, respectively. Bivariate analysis was used to assess variable associations. A p-value of <0.05 was considered statistically significant. Receiver operating characteristic (ROC) curves were used to determine sensitivity and specificity, and logistic regression models were used to calculate odds ratios (OR) and 95% confidence intervals (CI) for predictive factors.

## Results

In the current study, total participants of 131 patients with symptomatic bladder outlet obstruction who underwent TURP were included. The median (IQR) of age among the participants was 71 (13) years.

BPH was detected in 66 patients (50.4%), while incidental prostate cancer (IPC) was detected in the other 65 patients (49.6%). Fifty patients with IPC in TURP specimens were classified as low-grade IPC according to the Gleason score, and the other 15 patients were classified as high-grade IPC. The demographic data of the patients were mentioned in Table 1.

**Table 1:** Demographic data among the studied patients (n=131) IQR, interquartile range; BMI, body mass index; DM, diabetes mellitus; HPN, hypertension; BPH, benign prostatic hyperplasia; IPC, incidental prostate cancer

Variable	BPH	Low-grade IPC	High-grade IPC	P-value
	(n=66)	(n=50)	(n=15)	
Age median (IQR)	71 (11.5)	70 (12.5)	76 (12.5)	
Age (N, %)				0.2
≤71 years	30 (45.5%)	27 (54%)	4 (26.7%)	
>71 years	36 (54.5%)	23 (46%)	11 (73.3%)	
BMI median (IQR)	27 (5.75)	28 (7)	27 (8.5)	0.9
BMI classification (N, %)				
Underweight	0 (0%)	2 (40%)	0 (0%)	0.7
Normal	17 (25.8%)	12 (24%)	3 (20%)	
Overweight	29 (43.9%)	19 (38%)	6 (40%)	
Obese	20 (30%)	17 (34%)	6 (40%)	
Comorbidities				
DM (N, %)	48 (72.7%)	11 (22.4%)	2 (14.3%)	0.6
HPN (N, %)	24 (36.4%)	17 (34%)	5 (33.3%)	0.9
Smoker (N, %)	32 (48.5%)	28 (56%)	10 (66.7%)	0.4
Alcoholic (N, %)	37 (56.1%)	31 (62%)	11 (73.3%)	0.4
Family history	8 (12.1%)	11 (22%)	3 (20%)	0.4

The IPC group had significantly higher PSA levels compared to the BPH group, and among the IPC group, the patients with high-grade IPC had higher PSA levels compared to low-grade IPC patients (p=0.003), as most of the patients with high-grade IPC (46.7%) had PSA levels of more than 10 ng/mL in comparison to 13.6% and 24% in BPH and low-grade IPC patients, respectively.

The weight of the resected specimens was significantly lower in high-grade IPC patients in comparison to low-grade IPC and BPH patients (p=0.03). A statistically significant difference was determined between low-grade IPC, high-grade IPC, and BPH, with a TURP weight of 17 g taken as the cutoff point (p=0.04).

NLR, PLR, and HALP scores were evaluated among the studied patients; there was a statistically significant difference in the NLR score (p=0.006) and a highly statistically significant difference in the HALP score (p<0.001) between the different studied groups (Table 2).

**Table 2:** Characteristics among studied patients (n=131) PSA, prostate-specific antigen; HALP, hemoglobin-albumin-lymphocyte-platelet; NLR, neutrophil-to-lymphocyte ratio; PLR, platelet-to-lymphocyte ratio; IQR, interquartile range; BPH, benign prostatic hyperplasia; IPC, incidental prostate cancer

Variable	BPH	Low-grade IPC	High-grade IPC	P-value
	(n=66)	(n=50)	(n=15)	
PSA median (IQR)	3 (5)	3.5 (6)	9 (18)	0.009
PSA (N, %)				
≤4 (ng/mL)	38 (57.6%)	33 (66%)	3 (20%)	0.003
4-10 (ng/mL)	19 (28.8%)	5 (10%)	5 (33.3%)	
>10 (ng/mL)	9 (13.6%)	12 (24%)	7 (46.7%)	
Specimen weight median (IQR)	19 (42.3)	17 (16)	10 (8)	0.03
Specimen weight (N, %)				
≤17 (g)	29 (43.9%)	23 (46%)	12 (80%)	0.04
>17 (g)	37 (56.1%)	27 (54%)	3 (20%)	
NLR score median (IQR)	2.79 (2.2)	3.78 (4.9)	6.71 (4.8)	0.006
NLR score (N, %)				
≤3.3	39 (59.1%)	23 (46%)	4 (26.7%)	0.05
>3.3	27 (40.9%)	27 (54%)	11 (73.3%)	
PLR score median (IQR)	128.35 (81.2)	155 (109.8)	151.7 (154.9)	0.2
PLR score (N, %)				
≤141.4	37 (56.1%)	23 (46%)	6 (40%)	0.4
>141.4	29 (43.9%)	27 (54%)	9 (60%)	
HALP score median (IQR)	31.25 (31.3)	27.5 (27.5)	17 (16.2)	<0.001
HALP score (N, %)				
≤0.33	19 (28.8%)	30 (60%)	14 (93.3%)	<0.001
>0.33	47 (71.2%)	20 (40%)	1 (6.7%)	
Submission of specimen				0.2
Representative	35 (53%)	20 (40%)	5 (33.3%)	
Entirely	31 (47%)	30 (60%)	10 (66.7%)	

**Figure 2:**
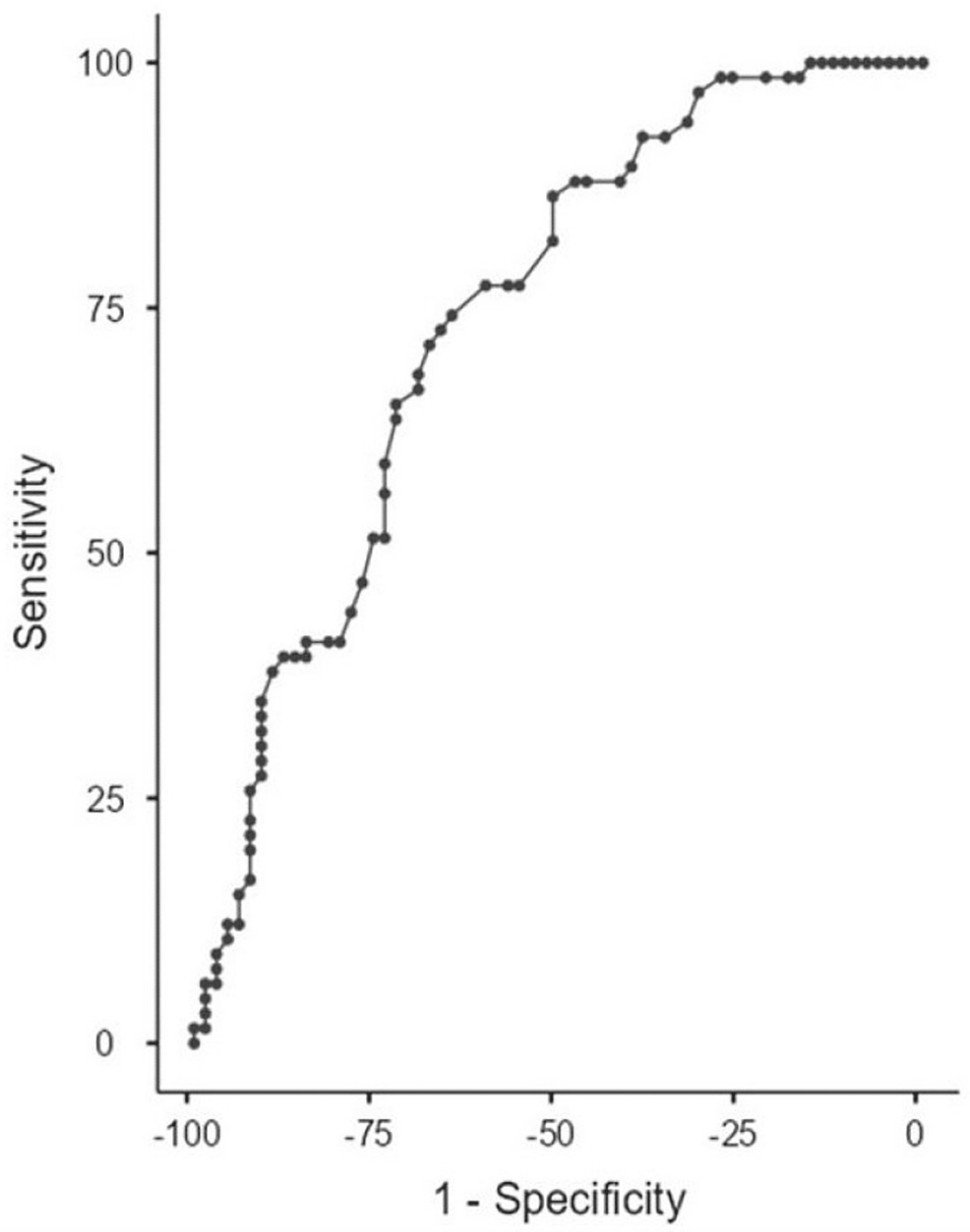
Receiver operating characteristic (ROC) curve analysis to discriminate between BPH and IPC BPH, benign prostatic hyperplasia; IPC, incidental prostate cancer

In ROC analysis, the area under the curve (AUC) for discriminating patients with BPH from those with IPC according to the HALP score was 0.738. According to the findings of the ROC analysis, the cutoff value for the HALP was set at 0.33. The sensitivity and specificity for the HALP score were 71.21% and 67.69%, respectively (Figure 2).

**Figure 3:**
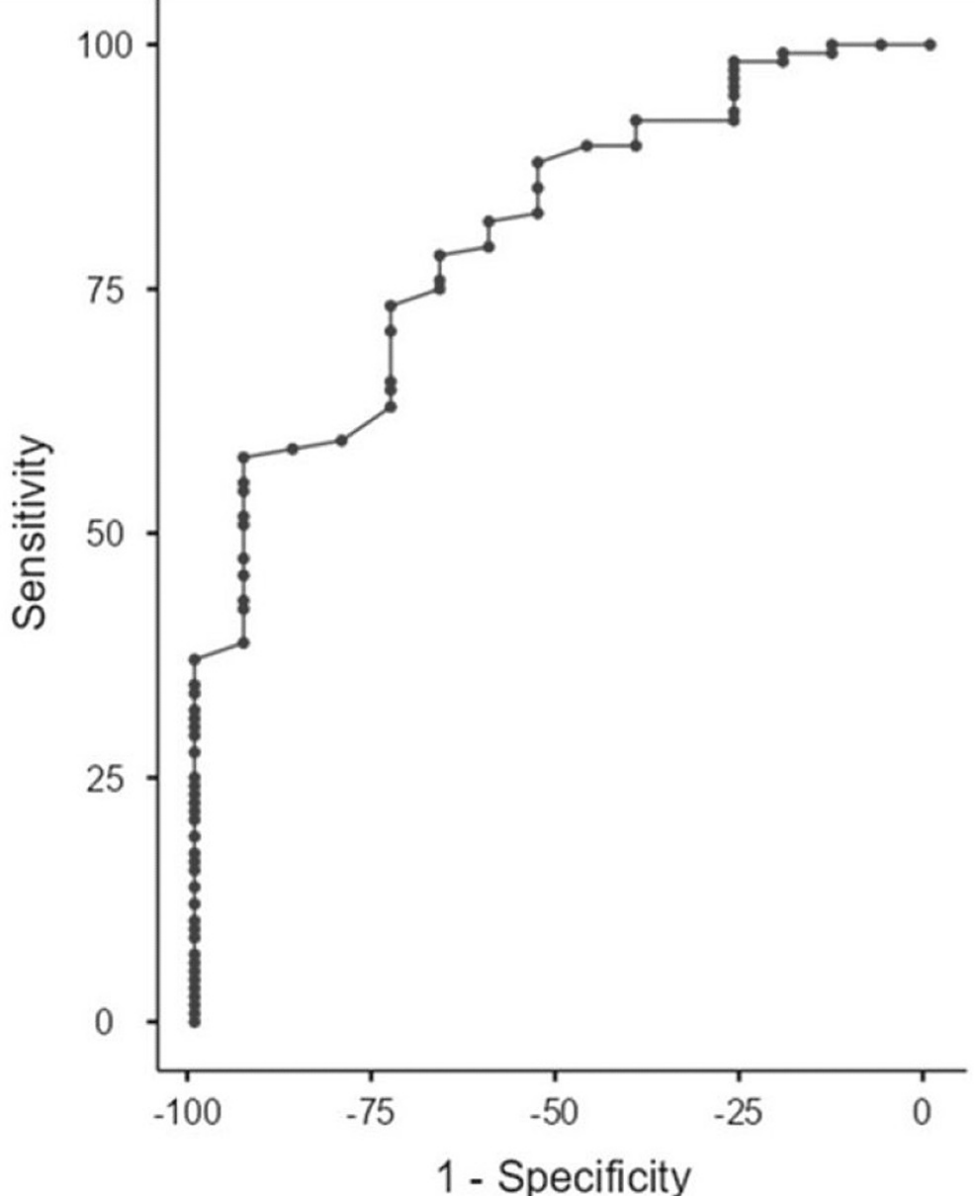
Receiver operating characteristic (ROC) curve analysis to discriminate between the benign prostatic hyperplasia (BPH) and incidental prostate cancer (IPC) analysis of the HALP score to discriminate high-grade from low-grade incidental prostate cancer (IPC) and benign prostatic hyperplasia (BPH) HALP: hemoglobin-albumin-lymphocyte-platelet

Another ROC analysis for discriminating patients with high-grade IPC from BPH and low-grade IPC according to the HALP score was performed. The AUC was 0.8 with a sensitivity of 55.17% and a specificity of 93.33% at a cutoff point of 0.34, so the HALP score could be considered a good discriminator between patients with high-grade IPC and those with BPH and low-grade IPC (p=0.004) (Figure 3).

To investigate the potential risk factors for IPC detection, we conducted logistic regression analyses using univariate and multivariate methods. In the univariate logistic regression, only PSA (odds ratio {OR}, 0.03; 95% confidence interval {CI}, 1.003-1.11; p=0.03) and the HALP score (OR, 0.96; 95% CI, 0.94-0.98) were significantly associated with IPC after TURP, whereas no effect of other predictors was observed. Interestingly, the present study indicated that increased PSA (OR, 1.09; CI, 1.02-1.16) and decreased HALP score (OR, 1.01; 95% CI, 0.002-0.13) were significant independent predictive factors for IPC after TURP. In addition, smaller specimen weight was associated with increased incidence of IPC (OR, 0.98; 95% CI, 0.97-0.99) after TURP (Tables 3, 4).

**Table 3:** Logistic regression analysis for the predictors of IPC IPC, incidental prostate cancer; PSA, prostate-specific antigen; HALP, hemoglobin-albumin-lymphocyte-platelet; NLR, neutrophil-to-lymphocyte ratio; PLR, platelet-to-lymphocyte ratio; OR, odds ratio; CI, confidence interval

Variable	IPC			
	Univariate		Multivariate	
	P-value	OR (95% CI)	P-value	OR (95% CI)
Age	0.3	1.02 (0.98-1.06)	0.2	1.03 (0.99-1.07)
PSA	0.03	1.05 (1.00-1.11)	0.02	1.09 (1.02-1.16)
NLR score	0.45	1.01 (0.99-1.03)	-	-
PLR score	0.16	1.00 (0.99-1.01)	-	-
HALP score	<0.001	0.96 (0.94-0.98)	<0.001	0.01 (0.002-0.13)
Specimen weight	0.19	0.99 (0.98-1)	0.007	0.98 (0.97-0.99)

**Table 4:** Pairwise comparison of the studied groups BPH, benign prostatic hyperplasia; IPC, incidental prostate cancer; PSA, prostate-specific antigen; NLR, neutrophil-to-lymphocyte ratio; HALP, hemoglobin-albumin-lymphocyte-platelet

Pairwise comparison	PSA	Specimen weight	NLR score	HALP score
	P-value	P-value	P-value	P-value
BPH to low-grade IPC	0.88	0.91	0.16	0.001
BPH to high-grade IPC	0.005	0.04	0.008	<0.001
Low-grade IPC to high-grade IPC	0.03	0.03	0.17	0.04

On conducting regression analysis for predictor factors of high-grade IPC, the HALP score was the only significant independent predictive factor associated with high-grade IPC (OR, 0.92; 95% CI, 0.87-0.97) (Table 5).

**Table 5:** Logistic regression analysis for the predictors of high-grade IPC IPC, incidental prostate cancer; PSA, prostate-specific antigen; HALP, hemoglobin-albumin-lymphocyte-platelet; NLR, neutrophil-to-lymphocyte ratio; PLR, platelet-to-lymphocyte ratio; OR, odds ratio; CI, confidence interval

Variable	High-grade IPC			
	Univariate		Multivariate	
	P-value	OR (95% CI)	P-value	OR (95% CI)
Age	0.07	1.06 (0.99-1.13)	0.06	1.07 (0.99-1.15)
PSA	0.08	1.01 (0.99-1.02)	0.28	1.01 (0.99-1.02)
NLR score	0.14	1.01 (0.99-1.03)	-	-
PLR score	0.12	1.00 (0.99-1.02)	-	-
HALP score	<0.001	0.92 (0.87-0.96)	0.004	0.92 (0.87-0.97)
Specimen weight	0.053	0.95 (0.89-1.001)	0.057	0.94 (0.88-1.00)

## Discussion

The global incidence of prostate cancer is on the rise. Current screening methods for prostate cancer involve a combination of digital rectal examination, elevated prostate-specific antigen (PSA) levels, and magnetic resonance imaging of the prostate [5].

However, these methods often yield false-positive results in various benign conditions such as prostatitis and BPH. Consequently, patients may undergo unnecessary biopsies or transurethral resection of the prostate (TURP), leading to unwarranted medical procedures, increased anxiety, and a heightened strain on healthcare systems.

Among these diagnostic methods, PSA testing garnered approval from the US Food and Drug Administration (USFDA) as a screening tool for prostate cancer in 1994. Presently, a PSA cutoff level of 4 ng/mL is conventionally employed for screening purposes. The incorporation of PSA screening has notably contributed to a substantial escalation in the detection of prostate cancer [6]. However, this advancement has concurrently resulted in an increased incidence of false-positive biopsies. This is attributed to the lack of specificity of PSA for prostate cancer, as elevated levels may be observed in benign conditions such as prostatitis and BPH. Furthermore, the limitations of PSA screening are evident in instances where PSA levels fall below the designated cutoff, resulting in missed detections in numerous cases of prostate cancer. Consequently, the exclusive reliance on PSA as a singular marker in screening practices has engendered a mixed perspective, owing to its susceptibility to false positives and the potential for false negatives.

In our investigation, we sought to assess the utility of the HALP score, which comprises hemoglobin, albumin, lymphocytes, and platelets, as a predictive tool for the occurrence of prostate cancer in patients undergoing TURP. Hemoglobin and albumin levels reflect nutritional status, while lymphocytes and platelets provide insights into the body's immune status, all of which have known implications in the initiation and progression of cancer. The HALP score has primarily been employed to predict the prognosis of various malignancies, including gastric carcinomas, esophageal squamous cell carcinoma, colorectal cancer, renal cell carcinoma, bladder cancer, lung cancer, and pancreatic cancer [6-11]. Furthermore, it has been explored as a prognostic indicator for metastatic prostate cancer post radical prostatectomy, demonstrating its independence as a prognostic factor [12]. Additionally, we computed two additional inflammatory markers, namely, the neutrophil-to-lymphocyte ratio (NLR) and platelet-to-lymphocyte ratio (PLR) scores, recognized for their impact on overall survival outcomes in individuals with prostate cancer. NLR is a marker of systemic inflammation and immune response. It is calculated by dividing the absolute neutrophil count by the absolute lymphocyte count obtained from a complete blood count (CBC) test. A higher NLR indicates an imbalance between neutrophils and lymphocytes, with elevated neutrophil levels relative to lymphocyte levels. This imbalance is often associated with increased inflammation and immune activation. Elevated NLR values have consistently been correlated with an unfavorable prognosis [13-17]. PLR is a biomarker that reflects the balance between platelet count and lymphocyte count in the blood. It is calculated by dividing the platelet count by the lymphocyte count obtained from the CBC test. PLR is increasingly recognized as a marker of systemic inflammation and immune response. Heightened PLR values have been linked to diminished overall survival rates [13-19].

In a precedent investigation addressing the assessment of preoperative HALP scores among individuals diagnosed with oligometastatic prostate cancer subsequent to cytoreductive radical prostatectomy, a noteworthy correlation was established [12]. Specifically, a low HALP score was significantly associated with a simultaneous reduction in progression-free survival. The utilization of the HALP score threshold set at 32.4 was integral to this analysis. Contrastingly, an alternative study focused on the application of HALP scores in the diagnostic context of prostate cancer failed to elucidate significant distinctions in HALP scores between cases of BPH and prostate cancer [20]. This particular investigation revealed that increased PSA levels, decreased HALP score, and smaller specimen weight were independent predictive factors for IPC after TURP. However, the HALP score was the only significant independent predictive factor associated with high-grade IPC. HALP scores within the prostate cancer group were higher compared to the BPH group. These findings underscore the nuanced role of HALP scores and the need for context-specific considerations in different clinical scenarios. Furthermore, our results showed that specimens with a smaller weight had a higher incidence of PC. There is a possibility that cancer may not be detected in larger-weight specimens. However, the clinical significance of undetected cancer is unlikely.

Our investigation revealed that the HALP score is an independent marker for the detection of prostate cancer. Patients with prostate cancer exhibited lower HALP scores in comparison to those with BPH. Notably, at a HALP score of ≤0.33, the sensitivity and specificity for detecting prostate cancer were 71.2% and 67.69%, respectively. Additionally, our study indicated that the HALP score could effectively differentiate high-grade prostate cancer from low-grade prostate cancer, achieving a specificity of 93.33%.

We acknowledge that there are several limitations that must be considered while interpreting our results. This is a single‐institution study, with a limited sample size despite identifying more than 330 patients initially. Due to the lack of relevant variables, large databases (such as National Cancer Data Base {NCDB} or Surveillance, Epidemiology, and End Results {SEER}) could not be used to answer the research question. The next step will be to perform a multicenter prospective study to further corroborate the findings of our study. Furthermore, the retrospective nature can only suggest associations rather than causation.

## Conclusions

This study found that among patients with symptomatic bladder outlet obstruction who underwent TURP, approximately half had BPH, while the other half had IPC. Patients with IPC, particularly those with high-grade IPC, had higher levels of PSA and lower resected specimen weight compared to patients with BPH. The HALP score showed promise as a discriminatory tool for distinguishing between BPH and IPC, as well as between high-grade IPC and BPH/low-grade IPC. Logistic regression analysis revealed that increased PSA levels, decreased HALP score, and smaller specimen weight were independent predictive factors for IPC after TURP. The HALP score was the only significant independent predictive factor associated with high-grade IPC. These findings contribute to our understanding of risk factors and diagnostic tools for IPC in patients with bladder outlet obstruction.
